# 53034 Melatonin use and occurrence of respiratory illnesses

**DOI:** 10.1017/cts.2021.481

**Published:** 2021-03-30

**Authors:** Hannah M. Bowen, Zachary A.P. Wintrob, Alice C. Ceacareanu

**Affiliations:** 1 Hartwick College; 2 ROAKETIN Inc.

## Abstract

ABSTRACT IMPACT: Melatonin use could alleviate virus-induced respiratory illnesses. OBJECTIVES/GOALS: Melatonin was identified as a potential repurposable drug in the fight against SARS Cov-2. Its ability to attenuate some virus inoculation effects raises the question whether melatonin use could alleviate virus-induced respiratory illness. Here we evaluated the occurrence of respiratory conditions in melatonin users and non-users surveyed. METHODS/STUDY POPULATION: Records from the Medical Panels Expenditure Survey (MEPS) database made available by the Agency for Healthcare Research and Quality were used to evaluate whether melatonin may be associated with reduced viral respiratory disease burden. First, all subjects reporting melatonin use (1996-2017) were collected along with records for all subjects reporting respiratory diseases as identified by consolidated ICD-9/10 codes. Second, all diagnosis codes were retrieved for all individuals identified in the first step. In total there were 201,490 occurrences of the specified conditions among 180,468 unique individuals. The relative risk of specific respiratory disease occurrence was computed for melatonin users and non-users. Population estimates for melatonin use were also determined. RESULTS/ANTICIPATED RESULTS: Among 221 melatonin users, 132 had at least one respiratory illness. Among the 180,468 total subjects reporting at least one respiratory condition, melatonin use was associated with a lower rate of the common cold, pharyngitis, strep throat, scarlet fever, and sinusitis. Furthermore, melatonin was associated with a significantly reduced risk of common cold (RR 0.760, CI 0.587-0.985) and sinusitis (RR 0.407, CI 0.186-0.890). Due to low subject counts, the reduced risk observed for scarlet fever and strep throat was not considered significant. Melatonin users had a higher relative risk of allergic rhinitis (RR 1.393, CI 1.043-1.862) and asthma (RR 2.166, CI 1.672-2.806), probably due to melatonin active prescribing in these patients as sleep aid. DISCUSSION/SIGNIFICANCE OF FINDINGS: Although melatonin showed a lower relative risk of certain viral respiratory conditions, the low melatonin user numbers and their heterogeneous distribution over the time interval led to highly variable population estimates. Yet, our data suggests that melatonin may alleviate viral respiratory illness and deserves further investigation.

